# Prolonged health worker strikes in Kenya- perspectives and experiences of frontline health managers and local communities in Kilifi County

**DOI:** 10.1186/s12939-020-1131-y

**Published:** 2020-02-10

**Authors:** Dennis Waithaka, Nancy Kagwanja, Jacinta Nzinga, Benjamin Tsofa, Hassan Leli, Christine Mataza, Amek Nyaguara, Philip Bejon, Lucy Gilson, Edwine Barasa, Sassy Molyneux

**Affiliations:** 1 Health Systems Research Group, KEMRI-Wellcome Trust Research Programme, Kilifi, Kenya; 2 Health Services Unit, KEMRI-Wellcome Trust Research Programme, Nairobi, Kenya; 3 Kilifi County Department of Health, Kilifi, Kenya; 4 Department of Epidemiology and Demography, KEMRI-Wellcome Trust Research Programme, Kilifi, Kenya; 5 Centre for Tropical Medicine and Global Health, Nuffield department of Medicine, University of Oxford, Oxford, UK; 6 Division of Health Policy and Systems, University of Cape Town, School of Public Health and Family Medicine, Cape Town, South Africa; 7 Global Health Department, Faculty of Public Health and Policy London School of Hygiene and Tropical Medicine, London, UK; 8 Health Economics Research Unit, KEMRI-Wellcome Trust Research Programme, Nairobi, Kenya

**Keywords:** Strike, Stressor, Conflict, Strategy, Frontline health managers, Community, Kenya

## Abstract

**Background:**

While health worker strikes are experienced globally, the effects can be worst in countries with infrastructural and resource challenges, weak institutional arrangements, underdeveloped organizational ethics codes, and unaffordable alternative options for the poor. In Kenya, there have been a series of public health worker strikes in the post devolution period. We explored the perceptions and experiences of frontline health managers and community members of the 2017 prolonged health workers’ strikes.

**Methods:**

We employed an embedded research approach in one county in the Kenyan Coast. We collected in-depth qualitative data through informal observations, reflective meetings, individual and group interviews and document reviews (*n* = 5), and analysed the data using a thematic approach. Individual interviews were held with frontline health managers (*n* = 26), and group interviews with community representatives (4 health facility committee member groups, and 4 broader community representative groups). Interviews were held during and immediately after the nurses’ strike.

**Findings:**

In the face of major health facility and service closures and disruptions, frontline health managers enacted a range of strategies to keep key services open, but many strategies were piecemeal, inconsistent and difficult to sustain. Interviewees reported huge negative health and financial strike impacts on local communities, and especially the poor. There is limited evidence of improved health system preparedness to cope with any future strikes.

**Conclusion:**

Strikes cannot be seen in isolation of the prevailing policy and health systems context. The 2017 prolonged strikes highlight the underlying and longer-term frustration amongst public sector health workers in Kenya. The health system exhibited properties of complex adaptive systems that are interdependent and interactive. Reactive responses within the public system and the use of private healthcare led to limited continued activity through the strike, but were not sufficient to confer resilience to the shock of the prolonged strikes. To minimise the negative effects of strikes when they occur, careful monitoring and advanced planning is needed. Planning should aim to ensure that emergency and other essential services are maintained, threats between staff are minimized, health worker demands are reasonable, and that governments respect and honor agreements.

## Introduction

### Health worker strikes in low and middle-income countries

Health worker strikes are experienced globally, but the effects have been argued to be worst in low and middle-income countries (LMICs) due to infrastructural and resource challenges, weak institutional arrangements, underdeveloped organizational ethics codes and practices, and lack of alternative available and affordable healthcare [1–3]. Strike actions range from merely halting work for a few hours or curtailing of non-critical services, to complete stoppage of work; with the latter usually reserved as the last resort [4–6]. A challenge in the health sector is that when health workers strike, there is potential to harm patients and the broader public. There is therefore always significant debate on the ethical issues associated with health worker strikes [1, 5, 7–18].

In any setting, strikes do not automatically impact on health outcomes such as mortality [19–21] or cause a total shut down of health service delivery [1, 22]. Rather, the effects depend on the length of the strike, the specific strike actions adopted, responses by the management and the ability of affected populations to access alternative care [1, 3, 19, 23]. In addition, in settings where healthcare provision under normal circumstances is particularly compromised, service provision or health outcomes may be minimally affected by health workers strikes; the norm cannot really get much worse [24]. Few studies have examined the experiences and impacts of strikes in LMICs, and globally most strike related studies have focused on the impact of strikes using mortality as a key indicator [3], or on the ethical issues emerging from strikes [1]. We are not aware of any published empirical studies that have explored responses and experiences of a strike from the perspectives of both frontline health managers and residents of local communities. Such an approach is valuable in contributing the voices of those who are arguably most affected by strikes when they occur, and in generating ideas on how to nurture health system resilience in the face of such shocks.

### A brief history of health worker strikes in Kenya

Kenya’s health system has in recent decades faced a range of chronic stressors including drug shortages, understaffing, and underfunding [25, 26]. In recent years two additional acute stressors or ‘shocks’ have been strikes and devolution [27, 28]. Between 2010 and 2016 there were six nation-wide strikes and many more regional strikes [24, 27]. General discontent and unrest among health workers appeared to coincide with the devolution of healthcare services in 2013, with claims that the devolution process was far more rushed than originally planned by technocrats [29] resulting in challenges with human resource management functions [25]. Health workers went on several strikes in protest, returning to work with their concerns largely unresolved, leading to further strikes [27, 30] a pattern that continued until 2016.

In 2017 there were two dramatic and prolonged health worker strikes: a 100 days doctors strike and, soon afterwards, a 150 days nurses strike; with the latter being the longest health worker strike in Kenya’s history. The official reason given for the prolonged nurses strike was failure by the government to sign and implement the nurses collective bargaining agreement (CBA) that had been agreed between the Kenya National Union of Nurses (KNUN) and the national and county governments in December 2016 following a two week nurses strike [31]. A CBA is a legally binding document aimed at fostering harmony between employer and employees by stipulating collectively agreed terms and conditions of service for employees [32]. The doctors and nurses CBAs included an agreed increment in allowances. While the doctors’ CBA was drafted much earlier and signed in 2013, it was not implemented until the 100 days strike [27]. The nurses had hoped to achieve the same with their strike, but 150 days in, the strike was suspended when the KNUN and the two levels of government agreed instead on an arrangement that would see the nurses receive their nursing service allowance in phases and an increase in uniform allowance by Kshs 5000 (50 USD) [31].

We explored the perceptions and experiences of frontline health managers and community members of the 2017 prolonged health worker strikes towards the end of the nurses’ strike and just after it had ended. Although there have been some publications documenting the impact of Kenyan health workers’ strike on mortality [24] and in-patient admissions [27], the perceptions and experiences of frontline health managers and community members have not been explored. In this paper, we draw on the perceptions and experiences of frontline health managers and community members to discuss strategies to minimise the potential for further strikes, reduce the negative effects of strikes when they occur, and nurture health system resilience more broadly.

## Methods

### Study setting

Kenya is a lower middle-income country with an estimated population of 45.1 million in 2015. 65% of the population reside in rural areas [33], and the country has an estimated poverty rate of 45.9% [34]. Since 2013, there has been a devolved system of governance structured around two administrative levels: national and county governments [35]. Within the health sector, the national government roles entail formulation of health policy and standards, and management of national referral services, while the 47 county governments have health service delivery roles [36, 37]. Counties are further divided into sub-counties which in the health sector are headed by sub-county health managers who form the link between the county health managers and facility health managers. The provision of healthcare is split evenly between public and private services [33], with the public sector organized across six levels: community services (level one); primary care (dispensaries and health centres – levels two and three); county referral services (levels four and five hospitals); and national referral health services (level six). Prior to devolution, level four hospitals were district hospitals while level five hospitals were provincial hospitals [38, 39]. There is heavy reliance on out-of-pocket payments which contributed 29% of total health expenditure in 2013 [33]. There is low insurance coverage at 19% [40], and high incidence of catastrophic household health expenditures at 6.58% [33].

### Study design

This study was conducted as part of our ‘learning site’ research [41]. Our learning site approach, currently being implemented in Kenya and South Africa, is a form of embedded health policy and system research (HPSR) in which we seek to co-produce knowledge with colleagues from the Kilifi County Department of Health [42]. This entails researchers and health managers working together to identify key health system research questions and interventions [41, 43, 44]. The approach includes sustained engagement with the managers to build mutual understanding, trust and enable access to tacit knowledge. Research activities within the learning site have focused on examining health governance at the sub-national level [41, 45]. To deepen our understanding of the perceptions and experiences of health managers, there are iterative cycles of data collection, analysis and interpretation, including reflection (within the research team and with managers) and action-learning [43, 46]. The learning site in Kenya is situated in Kilifi county. Kilifi county has an estimated population of 1.5 million people [47], with 68% living below the poverty line [48]. Table 1 presents the key demographic and health indicators of the county.

**Table 1:** Key demographic and health indicators in Kilifi county [47]

Indicator	Kilifi County 2018
Population estimates	
Total Population	1,498,647
Male	723,204
Female	775,403
Under 5 years	259,538
Under 1 year	54,518
Health personnel (public)	
Nurses per 10,000 people	4
Doctors per 10, 000 people	1
Health Facilities	
Public	143
Non-governmental	9
Faith-based	13
Private for profit	135

### Data collection procedures

Learning sites are considered an embedded form of HPSR due to the engagement sustained over a long period [42, 49], and our data were collected as part of learning site activities. Data collection methods within the learning site included informal observations, informal interviews, and regular reflections on learning among the research team members, and with health managers [49]. Formal in-depth interviews and document reviews which focused specifically on the experiences during the strike period were also held during, or soon after the end of the nurses’ strike. Although we set out to explore the interviewee’s perceptions on the nurses’ strike, these views were influenced by (and often intertwined with) experiences of other health worker strikes, and particularly the prolonged doctors’ strike which preceded the nurses’ strike. We did not include the views of frontline staff and private facility managers and identify this in the discussion as a potential limitation to our work.

For the health manager interviews, we purposively selected county and sub-county health managers, hospital managers from the three main public hospitals in the county, and frontline managers from four PHC facilities (two rural and two urban). Selection was aimed at exploring the range of perceptions and experiences in each case, and interviews were primarily individual in-depth interviews lasting 45 min to 1 h. A total of 26 individual interviews were conducted with health managers (Table 2). Topics addressed in the interview guide included perceptions on the causes and impacts of the prolonged strikes, strike-related stresses for facility, county and sub-county level health managers, and health managers’ responses to these stresses and questions on the implications for service delivery, the broader health system and public health (Additional file 1).

**Table 2:** Data collection and sampling

Data collection method	Purposively selected study participants	Sample size
In-depth interviews	Senior managers	5
	County health managers	
	Middle level managers	6
	Sub-county health managers	
	Hospital managers	9
	Primary healthcare facility level respondents	4
	Health-centre in charges	
	Dispensary in-charges	2
Focus Group Discussions	Facility management committee groups (FMC)	4 groups (total 20 individuals)
	KEMRI Community representative groups (KCR)	4 groups (total 36 individuals)

To document community perceptions, we held focus group discussions (FGDs) with community representatives on the facility management committees of the four selected PHC facilities, and with four groups of KEMRI community representatives (KCRs) (4–10 people per FGD; total 56 participants; Table 2). KCRs are community members elected by the community to interact with researchers at our research institution, the KEMRI-Wellcome Trust Research Programme (KWTRP). They are typical members of the community in the geographical area they work in as opposed to local leaders or spokespeople [50]. Facility committee members are positioned at the interface between the community and service delivery and so can offer perspectives and experiences at both the health system and community or household levels. FGDs lasted approximately one hour and forty minutes, focusing on: perceived causes and impacts of the strikes; implications for treatment-seeking, service delivery, costs and quality of health care received, and health outcomes (Additional file 2).

Interviews and FGDs were conducted until we reached a point of saturation where no new information was being generated [51].

### Document review and use of existing data

We reviewed 5 sets of documents identified online and through interviews with information on the causes or effects of the 2017 nurses strike, and on efforts to keep services running. These included: the 2013 and 2016 CBA agreements; KNUN meeting minutes and memos; the 2013 Return to Work Formula for Health Workers; and the District Health Information System 2 (DHIS2) database (a nation-wide web-based reporting system capturing facility-level monthly service utilization data) [52]. DW and NK manually extracted information on the history, causes, prolonged nature and effects of the strikes from the documents listed. Data from interviews, observations and documents were complemented by data from the Kilifi Health Demographic Surveillance System (KHDSS) which records hospital admissions, births, pregnancies, migration events and deaths of approximately 300, 000 people in the county through 4-monthly household visits [48]. DW and AN extracted data on outpatient in-patient and service utilization during the strike periods from the DHIS2 and KHDSS databases respectively.

### Data management and analysis

For the qualitative data, we adopted a thematic analysis approach as described by Braun and Clarke [53]. DW and NK developed detailed summaries for each discussion or observation period, guided by the topic guides, interview data, field notes and debriefs. Recorded interviews were transcribed, entered into NVivo Version 12 and coded. Coded data and detailed summaries were then drawn upon to fill charts based on our research/interview questions, and emerging learning (thereby combining deductive and inductive elements). Data from document reviews were also added into charts and provided wider contextual information. To support our analysis and shared learning, we held formal reflective practice sessions among the research team and together with health managers. These sessions allowed for some ‘member checking’ [54] on credibility of findings and resonance with diverse experiences.

Quantitative data obtained from the two databases (DHIS2 and KHDSS) were used to draw graphs (using R with ggplot2 package) [55, 56] showing trends in outpatient and in-patient service utilization and how these were affected during the strike periods.

## Results

Following our interviewee’s perspectives on the causes of the strike and its’ length, we present data on the overall effects of the strikes at health system and household levels. We then present the activities health managers undertook to cope with those disruptions. Table 3 below presents a summary of the main themes and related content identified.

**Table 3:** Summary of identified themes and subthemes

Theme: Perceived causes of the prolonged 2017 strikes	Theme: Overall perceived effects of the strikes	Theme: Efforts to keep services running
*-Frustration with government’s failure to honour previous CBAs*	*Disruptions of service delivery* -Decline in in-patient admissions -Reduction in outpatient service utilisation	*Prioritization of specific services* -Prioritization of emergency and chronic care services
-Simmering discontent with *human resource processes and poor working conditions*	*Households missed care with related health outcomes* -Delays in accessing care due to uncertainty about which public facilities were open -Reported health related effects included: unwanted pregnancies, maternal complications and deaths, new-born deaths and long-term complications from delayed treatment	*Minimising and managing conflict* -Nurse managers tried to calm tensions and conflict between striking and non-striking nurses *Drawing on NGO staff, other cadres and students* -NGO staff continued to offer services in NGO supported areas such as TB/HIV -In some PHC facilities, support staff reportedly dispensed medications for minor ailments -Task-shifting to students and other non-striking cadres
*-Differences and unfairnesses across cadres of health workers*	*Alternative sources of care and increased costs of care:* - Private facilities were an alternative source of care -Other reported alternatives include use of herbs or self-medication -Some households had to borrow or sell household assets to meet costs of care in private facilities	*Links and interactions with private facilities:* -Adoption of an informal system of transferring patients for post-operative care in private facilities during both strike periods. -Sub-county managers increased supervisory visits to private facilities and provided these facilities with supplies to ensure community members continued to receive services.
*Broader political context & activities* -Politics reportedly took precedence over negotiation with nurses as politicians and government actors focused on national and county elections -Poor co-ordination between national and local governments impacted handling the strike	*Demotivation among health system staff:* -Health managers reported working long hours and feeling unsupported by their supervisors -Service provision was slower and more tasking for the non-striking staff leading to demotivation *Loss of trust in the public health system:* -Service disruption caused by high recurrence of strikes contributed to loss of trust in the public sector among community members	*Support and action from the public:* -Community members reportedly supported the health workers’ strikes. -However, after the death of two pregnant women during the nurses’ strike, some urban residents engaged the media to protest the continued strike. -Other community members almost burned down a private facility for demanding payment to remove the body of a woman who had been denied maternity care due to lack of funds

*CBA* Collective Bargaining Agreement, *HIV* Human Immuno-Deficiency Virus, *NGO* Non-Governmental Organisation, *PHC* Primary Health Care, *TB* Tuberculosis

## Perceived causes of the PROLONEGD 2017 nurses strike

Most interviewees echoed the official reason for the strike in stating that nurses wanted the government to honor its promise of signing their CBA to increase their pay and improve working conditions. The frustration of not having the CBA signed and implemented was reportedly compounded by the Salaries and Remunerations Commission (which advises government on public officer’s remuneration and benefits) defining nurses as semi-skilled and thus only eligible for relatively low pay scales, and doctors having had their CBA implemented when they went on strike:

*“So, people were demoralized, they were like there was discrimination: other cadres are important [while] others are not important; some non-professional, others are professional. So, with that people decided we need to be recognized, let us go on strike and never come back unless our CBA is signed.” Hospital Manager-03.*

Poor working conditions, including shortages of drugs, commodities, equipment and staff, were also felt to have contributed to demoralization among nurses, with several managers noting that these challenges had been exacerbated through policy changes such as devolution, free maternity care policy, and abolition of outpatient user fees at PHCs:

*“We say that maternity services are free, yet the same facilities are not facilitated with the commodities. So, there is a gap such that the outcome of the mother and the child is 50-50 so because of that we are human beings and sometimes it’s not good always to see that your patients are dying in your hands. You would rather not provide that service.” Sub-County Manager-06.*

Previous county level agreements on for example promotions, re-designations into appropriate job groups, and training had only partially been implemented, and therefore failed to address the general discontent and unrest among health workers.

A lack of clarity and agreement between national and county levels on how to handle national-wide strikes was seen to have contributed to the prolonged length of the strike:

*“The central government used to accuse the county government. The county government … blame the central government that they have not disbursed the money … so there was a lot of shifting blames.” Sub-County Manager-06.*

The nurses’ strike took place during a fevered election campaign period for national president and members of parliament and county leadership. With the outcome of the August 2017 presidential elections disputed and later annulled by Kenya’s supreme court and fresh elections having to be organised, politics reportedly took precedence over service delivery. Both sides -nurses and the government– then also took a hard stance: the nurses wanted their CBA demands met before they resumed duties, while the government considered the nurses’ CBA demands too costly. To handle this impasse, the government gave ultimatums and described the strike as illegal, possibly breeding further resistance.

Some respondents - particularly community members - felt that an underlying issue was that those in government who should have been working to solve the issue did not prioritise it because they could easily afford private care; highlighting feelings of injustice and distrust. Notably, at the time of our interviews - conducted immediately after the strike - the nurses had not received all the agreed allowances, and their CBA had not been signed. Most respondents felt that we should *‘expect more strikes’*, by nurses and other cadres.

*“Yeah, we expect more strikes very soon because if you give one son [doctors] and deny another one [nurses] then expect more strikes very soon unless the government sits down and thinks about this issue …*. *They are not being given their rightful share as simple as that.” Hospital Manager-04.*

## Overall perceived effects of the strikes

### Facility and service related disruptions and its effects

All public facilities in the county were severely disrupted by the health worker strikes, although the nature of the disruptions differed by facility level. During, the nurses’ strike, most dispensaries –primarily run by nurses – were closed, and many health centres ran on a ‘go-slow’ basis with nurse-led services such as maternity, maternal child health and child welfare clinics were particularly affected.

*“The most that were affected mainly are the dispensaries because they were mainly run by the nurses’ fraternity, yeah …*. *[most] of the dispensaries were totally grounded … the health centers you could find other cadres like the clinicals officers [but even there] it looked like a go-slow because you could take an hour before you were seen …*. [*the dispensaries] were totally … kaput unless there were nurses who were on temporary basis who could come in and assist.” Sub-County Manager-06.*

This disruption of service delivery in PHC facilities also negatively influenced preventive and promotive community-based services coordinated by community-based health workers (CHWs) from facilities. There was also a reported decline in hospital admissions and theatre services given the interdependence across cadres at hospital level:

“*… the health system is a group of around 18 different cadres. Once you try to interfere with just one [the nurses] you have affected the whole of it … the system is interdependent, so you still end up affecting the whole system.” Sub-County Manager-05.*

**Fig. 1:**
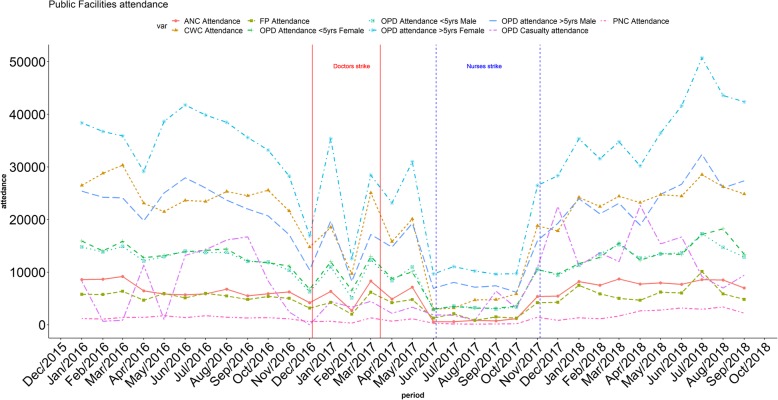
Outpatient services in Public Facilities showing sustained decline in services during the nurses' strike

**Fig. 2:**
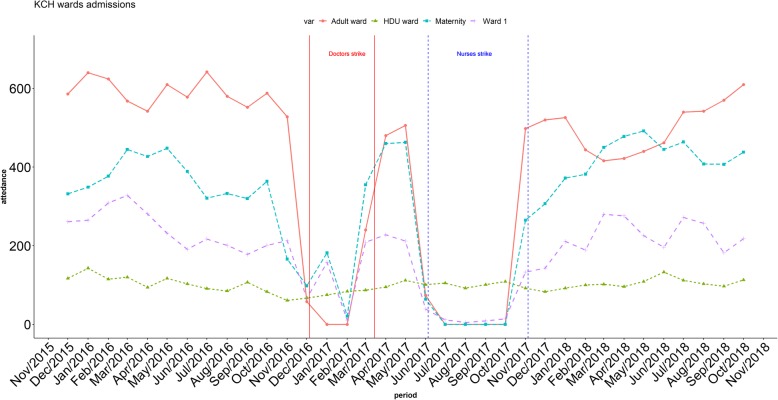
In-patient admissions at Kilifi County Hospital showing decline in admissions during both the doctors' and nurses' strikes

Utilisation records support findings that many key services were markedly reduced (Figs. 1 and 2). During the earlier doctors’ strike, outpatient facilities remained open (Fig. 1), perhaps due to the presence of nurses and clinical officers, but inpatient services were severely impacted, with few admissions if any (Fig. 2). During the nurses’ strike, both outpatient and inpatient facilities were impacted. The paediatric High Dependency Unit (HDU) run by the KWTRP remained operational during both strike periods which may explain the absence of variation in paediatric admissions.

Facilities were also affected by expired drugs and other commodities having to be destroyed. Some respondents mentioned that the prolonged 2017 health workers’ strikes contributed to strengthening of the private sector vis a vis the public sector:

*“in fact we created room for mushrooming of private facilities. For instance now you drive down government road and you see a beautiful pink facility there. They are tapping on a resource and people who couldn’t get their services here [at government facility]. So, we are selling ourselves.” Hospital Manager-05.*

### Households missed care with health-related outcomes

At the community level there was a lack of clarity on what public sector services were open and when, and an assumption among community members that if one cadre of health workers was on strike, all facility services would be closed:

*“You know outside there, anybody is a doctor. When they are told there is a strike, they don’t come, they go to look for where the doctor is, so that confidence in the facility went down.” Hospital Manger-09.*

Interviewees reported significant delays in community members accessing health care due to the time spent looking for services, moving between public and private facilities, and negotiating initial deposits at private facilities. Many interviewees talked of ‘*people suffering so much and many dying’*. Physical health-related effects reported included an increase in maternal and newborn deaths, long-term complications resulting from no or inadequate treatment, and large numbers of unwanted pregnancies due to poor access to family planning services. Respondents anticipated long term health-related consequences for those missing TB/HIV drugs, vaccinations, antenatal care, and skilled birth attendant deliveries.

*“Majority of the indicators are moved by the nurses; immunization, antenatal, family planning and deliveries. So, you really feel for these mothers, they have been at home for all this time, waiting for us. Because there were very few [mothers] who managed to go to the private facilities. Then the other thing which I really feel we might very soon start seeing diseases of the past coming back. Because majority of our community have not been immunized. As much as we have the doctors, the primary health care if not worked on well then you expect chaos in that particular community.” Sub-County Manager-04.*

### Households sought alternative sources of care and faced increased costs

To cope with the widespread closures and uncertainty of public sector services, many households reportedly turned to local private facilities or healers, or – for those unable to raise the required funds, or living remotely - started to depend more heavily on self-medication with shop bought drugs or herbs.

*“Now you will find it [strike] also taught people on how to look for alternative ways to coming to hospital, and if it continues that way, others might harm themselves with traditional herbs outside there”. Facility Management Committee FGD-03.*

To access funds for alternate care in private facilities, many community respondents reported that household members had to fund raise and borrow funds, or sell off precious assets such as domestic animals. For some of these households the effects of trying to meet these costs were described as catastrophic:

*“We brought another woman there [at a local private facility] she was forced to sell her land. The expenses were at 27000 [Kshs] within a week. She [even] wanted to sell her house, and then she died there and we couldn’t remove the body until you pay.” KEMRI Community Reprensentative FGD-03.*

### Health system staff felt guilty and demotivated

Most managers found the situation exhausting and stressful, working longer hours, taking work home, and feeling anxious about where their patients were going. Middle level managers’ frustrations were compounded by receiving pressure, and little support, from above.

*“It was not easy … from the county who were my supervisors, who I thought maybe they would come, see the situation on the ground, sit with actually me or us, deliberate on the issues, come up with possible solutions, and how they are going to support us, it never happened... Nobody came actually to find out, but they were asking for reports on daily basis until at a point I said, ‘No, I’m not giving any report to anybody and if people are willing to come let them come on the ground and find the report on the whatever is the situation on the ground.’ Hospital Manager-03.*

The strikes were also reported to have had negative effects on other actors within the system. For example, when one cadre was out on strike, the remaining cadres were demotivated by being unable to perform their roles as usual. It was reported that both doctors and nurses missed salaries for several months and that many suffered guilt and an internal tension between adhering to their oath to do no harm to the patients but at the same time feeling obliged to fight for their interests and stand with their colleagues.

### Loss of trust in the public health system

Several interviewees felt that the recurrence of strikes in the public sector and the disruptions and uncertainties brought by the prolonged strikes had contributed to a ‘*loss of trust’* from the public in the public sector that might have longer term effects in treatment-seeking and ultimately the public health system.

*“No, not everyone has managed to come back [to the public facility], some maybe they are still going to the private and they will still go. They have had bad experiences here during the strikes and this makes somebody lose trust in the government facilities. We wish our clients could come back.” Peripheral Facility Manager-06.*

## Efforts to keep services running

In the face of major facility and service closures and disruptions, frontline managers enacted a range of strategies in their efforts to keep services open, and re-open others. Across these strategies we observe some positive relationships, alliances and effects across the system, but also some tensions and conflicts. Many of the strategies were piecemeal, inconsistent and difficult to sustain.

### Prioritizing specific services and creatively using available health workers

At hospital level, emergency services were prioritized, but even these services, were difficult to sustain with depleted human resources:

*“during the doctor’s strike she [the facility manager] had to make sure there were a few nurses, and that the nurses would actually keep the place running* … *during the nurses’ strike we were doing emergency services both medical and surgical. But it was very strained because we didn’t have enough nurses to be able to monitor these post-operative patients. So, at some point we had to stop doing operations unless it’s a life-threatening case, so we had to turn away mothers. It was very painful.” Hospital Manager-07.*

During the nurses’ strike, staff in PHC facilities were keen to offer some outpatient chronic disease management services, particularly refills for HIV and TB patients, given the potential harms to patients and communities with treatment breaks. Middle level managers drew on available nurses in their efforts to offer priority services. Some were full-time nurses who opted not to strike, and others were probation or local contract nurses employed on terms that did not allow them to strike. Some of these staff were redeployed from PHC facilities to assist in managing emergency services at the hospitals.

Managers were also able to persuade some striking nurses to assist by appealing to their sense of duty:

*“We were telling them...we understand the plight of the strike … [but] … at the end of the day you are striking; the people who are affected is just the local person. And it’s them [the local people] being the victims while the people you are fighting against they are not feeling it.” Sub-county Manager-01.*

In one hospital (A) during the doctors’ strike, some Non-Government Organization (NGO) and privately practicing doctors were called upon to conduct emergency surgeries. The privately practicing doctors were engaged on locum basis (paid per number of hours/days worked), however, the arrangement did not last long as their payments were often delayed or missed out altogether. A similar strategy was employed during the nurses’ strike: nurses were brought in on a locum basis to assist in emergency services, but this arrangement only lasted for a few weeks (8-12 weeks) due to non-payment. More widely, some of the striking nurses were persuaded to assist with (NGO) sponsored health outreaches and services, by their managers. They were offered lunch and transport allowances to participate in these activities. These financial incentives were appreciated by nurses: they had their salaries stopped several months into the strike, and some were growing worried about their livelihoods in the face of no progress towards a strike resolution. Recognising the acute shortage of nursing staff, new short-term nursing staff were employed by the county government after several months.

### Minimising and managing conflict

During the nurses’ strike, nurses who assisted through the strike reportedly faced threats and intimidation from striking colleagues, who felt that their power was reduced by colleagues working. This resulted in some nurses withdrawing their help, or offering services inconsistently or secretly.

*“Some of us like me I was half strike half job. I would come peep and then I go. If I saw there were two or three patients that are very sick, I would treat them and run very fast so that the other nurses cannot find me here and beat me.” Peripheral Facility Manager-01.*

Tension between striking and non-striking nurses led to striking nurses reportedly sending non-emergency cases to a relatively well-resourced hospital to overwhelm their working colleagues. The hospital nurse manager had to intervene and calm the situation by informing them that everyone has a right to strike or not to strike:

“*… there was a division, those who were on, and those who were out. It was seen as a betrayal, the ones who were on duty it’s like they were betraying the others. So, I went to their solidarity corner and I told them it is your right to go on strike and it is their right not to go on strike, so everyone to play the ball in his own court and it calmed down and we continued offering services because before I addressed them when they saw each other they were abusing each other live [out loud]. Hospital Manager-02.*

### Drawing on NGO staff, other staff cadres and students

NGO employed staff did not seem to face the same pressures to join the healthcare workers’ strikes, and some NGOs employed additional staff to cover services such as HIV and TB in PHC facilities and hospitals. Interestingly, in one hospital, an NGO tried to pay striking nurses to work on locum basis but those nurses refused, possibly under pressure from their colleagues.

Given the challenges of finding adequate health workers to support services, there was some reliance across the system on support staff (staff not involved in direct patient care) and other cadres. For example, support staff in some PHC facilities were dispensing drugs for minor ailments and refills for TB and HIV clients during the nurses’ strike. In one subcounty, public health officers were instructed to offer immunization services (usually run by nurses). In one hospital (B), the nurse manager negotiated with a local colleague to let their nursing students remain at the hospital newborn unit under the supervision of the nursing in-charge. Also in hospital B, managers allowed clinical officers (non-physician authorized to perform routine medical duties and procedures) who had additional qualifications in reproductive health to conduct Caesarian Sections during the doctors’ strike. During the nurses’ strike, clinical officers were also allowed to take on postoperative nursing care under the oversight of the hospital nurse manager. However, over time there were concerns that the quality of care was being compromised and the obstetrics and gynecology consultant stopped the initiative.

### Links and interactions with private facilities

The above strategies were inadequate to fill the gaps, and so middle level managers used creative strategies to support access to services through local private facilities. For example, all three hospitals developed an informal system whereby they would perform emergency caesarean sections at the public hospital and have patients taken to local private facilities for post-operative nursing care. A similar strategy had been employed during the doctors’ strike where doctors working in the private-for-profit and NGO sector performed emergency caesarean sections in public hospitals and the public-sector nurses provided post-operative care. This pattern was to ensure the more expensive operations were performed at the public facility, and the cheaper nursing care services at private facilities, and therefore that patients were protected from catastrophic costs.

Subcounty managers also increased their supervisory visits to private facilities with an emphasis more on advice for handling volumes of patients rather than being too strict on quality control. Further, in some sub-counties, the managers supplied family planning and vaccines to local private clinics to ensure the services continued.

Although managers considered interactions with private facilities as necessary to support patients, there were concerns raised about the quality of care offered (particularly given increased volumes of patients), and the costs to patients:

*“We partnered now … we used the [private facilities] ...although now some started charging a lot of money [for family planning and vaccines they were getting freely]. We had to stop them again, renegotiate with them but we had no option, that was the only way to go to make sure that the services were continued.” Sub-County Manager-05.*

Another challenge was developing formal agreements. The county had many outstanding debts with private facilities from the doctors’ strike. In fact, some interviewees even felt private facilities might have been overly hiking the costs of caesarean sections to recoup some of their previous losses.

### Support and action from the public

It was noted that there was relatively little protest and action by community members to keep public services open. One reason might be that many community members reportedly supported the need for health workers to strike:

*“Even me I support them (striking nurses). The thing is everyone should respect another person’s work. If your colleague feels that he is suffering and is asking for his right, he should be given according to his education level and his work... Should he continue to suffer because people are dying? He shouldn’t claim for his right because he took an oath? That is not possible, because that is your work place... that is where he gets his daily bread.” KEMRI Comunity Representative-FGD04.*

However, the perceived inappropriate handling of the strike by government leaders who could better afford private care contributed to feelings of frustration and disillusionment in the community:

*“So, the good thing is in such situations [strikes], they should take them [nurses] seriously, and they should sit down, talk and give results which are helpful. ..Here in Kenya there are some counties that didn’t go on strike at all because their [county] government served them [the nurses] the way they wanted.” KEMRI Community Representative-FGD04.*

There were some community protests and actions. For instance, after the death of two pregnant women perceived to be due to difficulties in accessing affordable maternity care, some urban residents demanded that their facility provide maternity services at the minimum, and engaged the media to support their cause. They were successful in persuading public sector health workers to attend to pregnant women in the latter stages of labour. In this case the chairman of the local health facility, an elected community representative, apparently felt responsible to speak out and advocate for the rights of the poorest women in the community.

*“As the chairman of this facility all these [people] are looking at me, now if I also will sit there and look down, that’s not right. There are some means and I’m still strong even if the strike was going on now. There’s a mother who lost her life somewhere here. [She was expecting] twins and she wanted emergency [care. Yet] we have experts here (facility)... we know they could be able to handle that situation. This is a special place, you know there are rich people here, poor, and there are the poor and the poorest. So we are looking at the poorest people to get [help]*. *.*. *and I’m sure that mother she wouldnt have died, but she died.” Facility Mangement Committee- FGD04.*

In another example, community members reported protesting against a local private facility that denied maternity services to a woman in labour due to lack of funds, only for her to die at the facility’s premises while those who brought her were looking for funds and the facility to demand for payment before the body was removed. Community members reported that the facility was nearly burned down.

## Discussion

Our exploration of the prolonged 2017 strikes in Kenya highlighted a wide range of negative experiences at both health system/service and community levels. This is despite considerable efforts by many middle level managers, and some community protests and activism, to keep some essential services running. Here we discuss the political and health systems context of the strikes, the reported effects of the strikes and the potential to learn from health managers challenges and initiatives in future. We conclude with recommendations based on our data to reduce the likelihood of such prolonged strikes and associated negative impacts in future.

The health worker strikes cannot be seen in isolation of the prevailing context. There were multiple interacting factors ranging from political changes, health system challenges and increasing unionization of health workers. The implementation of a new devolved government in Kenya in 2013 made public sector workers’ right to unionize more explicit, except for the police and army [27, 57–59]. Meanwhile, longstanding issues including dissatisfaction with pay, working conditions and human resource management issues remained unresolved [24, 25, 27, 30]. When doctors and nurses did go on strike, the doctors’ CBA was signed but the nurses’ CBA was not. Such background conditions, and in particular feelings of unfairness between cadres, has also triggered strikes in Ghana and Nigeria [6, 60]. In Ghana, for example, strikes were called when doctors (but not other health workers) began being given additional duty hours allowances and – later – when the government decided to integrate these allowances into salaries (leading to higher pay scales for doctors than other health workers) [6].

Health systems exhibit features of complex adaptive systems (CAS) which include self-organization, feedback loops and emergence [61, 62]. Self-organization is a process in which the components of the system interact to create new states (termed emergence) which are often unpredictable or unintended [62, 63]. As CAS, the health system has separate parts, but these are interconnected and governed by feedback, and almost always, a change in one part, has effects on another part [62, 64]. Parallels can be drawn between the duty allowance saga in Ghana [6] and the cycle of strikes culminating in the 2017 strikes in Kenya. In both contexts, the governments failed to recognize the complex adaptive nature of health systems [61], by giving allowances to one group of doctors (Ghana) and by implementing only the doctors’ CBA (Kenya). Both the Ghanian and Kenyan governments adopted reactive responses, acting only when health workers went on strike as opposed to more proactively. In Kenya, county and national governments issued threats and ultimatums which did not solve the underlying problem. In both Ghana and Kenya, systematic analysis of possible reactions to interventions and consideration of context, power and interests of different actors may well have led to less dramatic impacts and shorter negotiation processes.

Also, important in the nature and length of the strikes, particularly the nurses’ strike, was the timing coinciding with national and local elections. Elections were already expected to be associated with unrest and to undermine the fragile public healthcare system [65]. While the timing may have been a strategy intended to add pressure on the government to meet the nurses demands, in fact it led to national and county leaders being distracted from the strike and its’ effects on patient and public safety. Our findings suggest a wide range of negative experiences. Disruptions to services and reduced admissions have also been documented by other studies by our group: one documented that the strikes resulted in marked reductions in admissions with 4 out of 13 county hospitals having almost no admissions throughout the strikes another found that the nurses strike severely affected immunization services in government-run referral health facilities across the country [27, 30]. Our finding of no obvious dip in outpatient service utilization during the doctors’ strike specifically is potentially linked to the presence of nurses and other cadres (such as clinical officers) in outpatients, but a forthcoming paper will characterize further the effect of both the nurses’ and doctors’ strikes on in-patient admission. Our interviewees highlighted the devastating effects of service disruption on staff morale and on households, particularly for the poorest households. Given that about 620,000 Kenyans are pushed below the national poverty line every year due to transport costs and health care payments even under ‘normal’ conditions [33], the impoverishing effect of the strike for the poorest households is likely to have been enormous. As with other sudden shocks to the health system [66], our findings support that the impoverishing effects of the strike are disproportionately felt by the poorest and most vulnerable*.*

Beyond impoverishment, interviewees talked in dramatic terms about negative health-outcomes linked to the strikes, including deaths, with the poor again being the worst affected. A recent analysis of the effects of six previous nation-wide Kenyan strikes on mortality data in Kilifi County (before the 100 days doctors and the 150 days nurses strike) found a 75% increase in mortality among children aged 12–59 months during the strike period, but no change in overall mortality [24]. The authors noted that the lack of change in overall mortality could have been because the strikes between 2010 and 2016 were relatively short, with only one lasting for more than a month (42 days). Evidence from other settings suggests that the effects of strikes on health outcomes are increased where emergency services are not available or the affected populations are not able to access viable (available and affordable) alternate healthcare services [1, 3, 19, 67, 68]. In Kenya, the Irimu et al (2018) study reviewing admissions in 13 public hospitals during the 2017 doctors’ and nurses strikes noted that ‘preventable deaths likely occurred on a massive scale’, particularly for the poor [27]. We identified similar perceptions in our study, but this may be in contrast with the more modest effects reported for prior strikes [24] . Given that the Kenyan public health system has faced a series of shocks and stressors over the decades, additional research that can provide more detailed data on the impact of the prolonged strikes on mortality over time is important.

An ‘everyday resilience’ lens is relevant for analyzing the strategies adopted by managers in response to strikes, and for considering the impact of the prolonged strikes on the Kenyan health system. Everyday resilience can be defined as the ability of the system to maintain positive adjustment in the context of chronic shocks and stressors in ways that allow the organization to emerge from those conditions strengthened and more resourceful [43]. Whether everyday resilience is observed and built in the face of chronic and acute stressors depends on the nature of the strategies enacted by health system actors, and the capacities that they can draw upon. Absorptive strategies buffer the system from shocks and return the system to its state with little or no change in structure; adaptive strategies result in some limited adjustments in the system structure or processes; while transformative strategies result in significant functional or structural changes [43, 69]. During the nurses’ strike in Kenya, we observed that middle level managers enacted a range of absorptive strategies in their efforts to keep services open, including mobilizing financial, infrastructural and human resources to support continuity of some essential services. Adaptive strategies included some reorganization of staff and services offered, but more significant functional or structural changes - transformative strategies - were not observed during the strike.

Across all the strategies observed, managers drew on their social networks and alliances to persuade and negotiate with various actors across the public health system to assist. They also demonstrated creativity in ways of working with others such as the local private facilities and NGOs. To keep key services running, managers drew on a long history of working together and coping with diverse everyday stressors in health service delivery [28, 41, 43]. Their relationships – or the ‘intangible resources’ they were able to draw upon - were sometimes invaluable in helping them cope with the shock of the strike. However, there was little to suggest that the broader system was undergoing positive adjustment to minimise the likelihood of future strikes or build preparedness in the event of any such strikes. Thus, there is little evidence that everyday resilience was being built over the course of the strike. Indeed, tensions between health system actors, including conflicts between striking and non-striking nurses (as also observed in South Africa [7], may have lasting negative implications for health system preparedness for and prevention of strikes. Our study did not include views from private facility health managers, but private facilities were frequently mentioned by community members and health managers as places where the public sought alternative care. A potential future research question might therefore be to examine if and how private providers can contribute to building resilience capacities that the health system can draw on in response to future strikes.

### Study limitations

We recognize that this study was only conducted in one county out of the 47 Kenyan counties, that interviewee’s perceptions of the nurses’ and doctors’ strikes were often intertwined and that we did not interview union officials, private health care providers and frontline health workers. Nevertheless, the trustworthiness of our data and analyses are supported through collection of data through several methods (interviews, observations, document reviews and surveillance data), allowing for some triangulation [68] of findings, member checking [54, 70] with researchers and health managers, and the use of an embedded approach that enhances trust between researchers and health system actors and hence the trustworthiness of collected data. Our sustained engagement with a wide range of respondents at county level and more widely across Kenya, supports the validity of our findings and their wider relevance.

### Conclusion and recommendations

The recurrence of health worker strikes and the prolonged nature of the 2017 strikes highlights the underlying frustration and unrest amongst public sector health workers in Kenya. There is an urgent need for national and county governments to appreciate the complex adaptive nature of health systems and adopt systematic monitoring of different components and proactive thinking around possible effects (positive and negative) of interventions and policies. County and national governments need to rebuild relationships with healthcare workers’ unions and include them in the development, introduction and implementation of policy decisions that impact on health workers [27]. Careful consideration is needed to review the compensation packages of health workers to ensure fairness within and across cadres, and the creation of a conducive working environment to offer quality services. This is essential to improving staff morale and reduce the need to strike.

Chima noted that an ethical approach to resolving labour disputes would require both employer and employee to recognize their moral obligation to serve public interest, reducing the incidence and effects of strikes [1]. An ethical approach to strikes is challenging to imagine in the context of political contest, constrained resources, great power imbalances between decision making actors and unclear public accountability. All parties should carefully consider the prevailing socio-economic and political circumstances when planning and managing a strike, including avoiding deliberately planning strikes around election times or in other times of predicted disruption and distress, particularly for the poor [7]. To ensure mutually respectful dialogue between parties, discussions may need to be facilitated by formal independent arbitration processes.

Recognising that strikes remain a real possibility, there needs to be adequate planning and preparedness in advance of a potential crisis [71]. Given their key intermediary roles, and the challenges they faced in the prolonged 2017 strikes, middle level managers should be better supported by managers higher up the system to design and implement effective and sustainable responses to sudden shocks, including strikes. Responses to shocks should not only seek to preserve core services but also to ensure that the poorest households and communities are protected from health-related and financial losses. This would support a move towards more ‘ethical’ strikes where at a minimum emergency and essential services are sustained throughout a strike, threats and intimidation of striking and non-striking health workers are minimized, demands by workers are reasonable, and governments respect and honor agreements.

## Supplementary information

**Additional file 1.** Topic guide for health manager’s interview.

**Additional file 2.** Topic guide for focus group discussion-community representatives.

## Data Availability

The data that support the findings of this study are available from KEMRI Wellcome Trust but restrictions apply to the availability of these data, which were used under license for the current study, and so are not publicly available. Data are however available from the authors upon reasonable request and with permission of KEMRI Wellcome Trust.
