# Supplementary material for: Prolonged health worker strikes in Kenya- perspectives and experiences of frontline health managers and local communities in Kilifi County
Source: Int J Equity Health. 2020 Feb 10;19:23. doi: 10.1186/s12939-020-1131-y (PMC7011250; doi:10.1186/s12939-020-1131-y)
Supplement: Supplementary file 1 — Additional file 1. Topic guide for health manager’s interview. [file 12939_2020_1131_MOESM1_ESM.docx]

**Additional file 1: TOPIC GUIDE FOR HEALTH MANAGER’S INTERVIEW-**

**Quick reminder about the consent form if that was shared in advance:**

- We are interested in the range of challenges faced by key people and organisations involved in the oversight of health delivery systems, and the strategies that managers use to respond to those challenges.
- This includes learning about what factors contribute to making organisations ‘resilient’, or able to withstand and even develop positively in a context of constant stresses (like understaffing) and sudden shocks (like disease outbreaks or big policy changes).
- We’d really value an opportunity to learn from you given your role and experience; your knowledge from within the system
- If you don’t mind, we’d like to record – only use information in general ways (show eg of policy brief)

**A bit about the interviewee: current role and responsibilities, who manages/managed by (formal line management and in practice), how long in current position, what doing before this, how did you end up in current role? Who are your team members?**

Overall, we recognize it’s been a challenging time for heath managers in the county this year, and possibly even since devolution. Before we go on to talk about some of the challenges, what have been some of the strengths/plusses to highlight? Anything you’d like to share with us about some of those?

Please tell us about some of what you think have been the most significant post devolution challenges? ….

- Eg related to priority setting, resource allocation, budgeting and financial management and human resource management.

**Thinking through some of the stressors/challenges, are there any other [think about/choose or three issues]?…**

- Which ones are challenges for you personally in conducting your role? Which ones are challenges for the system more widely? Can you give some examples?
- Is it possible to separate out the most pressing challenges/stressors from others? Do they influence each other?
- At your level in the health system what was done in response to the above issues to keep services up and running?

**Taking some of the most pressing challenges/stressors (including but not limited to the strike – all questions focus here on strike but can be amended as needed, recognizing we do want to focus on the strike):**

- More about the challenge and what’s brought it about: eg with the nurses’ strike:
  - *Summarise what we know about the strikes… which ones affected which cadres from when*
  - What do you think led to the strikes?
  - Why do you think this nurses’ strike went on so long? 5 months? How did it get resolved?
- At your level in the health system what was done in response to the strikes to keep services up and running? What about at other levels? What systems and structures were drawn upon by whom? Did new ones have to be developed? Formally/informally? Were you involved? How?
  - Eg in relation to prioritizing which services and activities (eg management meetings etc) to keep open/running at each of those levels? Which ones to close? How to change those being kept running?
  - Eg in relation to recruiting or reorganizing staff and their roles?
  - Eg in relation to public sector interaction with the private sector (eg any changes in who delivers what services, amount of referral, in which private facilities are registered with NHIF etc.)
- Any examples services/functions that were able to continue even in the face of the strikes? Gather examples. What facilitated this?
- What would you have liked to see happen to keep services up and running that didn’t happen or failed? *(gather detailed egs of efforts and why failed).*
- What has been the impact of the strikes on county health system so far? (county/sub-county/facility/ level). On you and your ability to do your job? On health system functioning more widely?
  - Eg Health workers and managers’ roles and motivation. Impacts more heavily on some cadres than others? Who and in what way?
  - Eg Patient/public access to prevention services? To health care? Ease of access? Quality? The costs they incur? Does this ‘hit’ some households more than others? Who and in what way? (depends on wealth, type of illness/service needed? Other? Gender? A combination?) e.g affordability and use
  - The interface between public and provide providers/facilities. More use of private facilities? Why/how with what impact
- What has given you and your colleagues strength/encouragement/support in dealing with challenges associated with the strikes?
  - Relationships with colleagues? Structures/processes in place? Mentorship, on-the job supervision? Other?
  - Any courses/training activities or any other interventions?
- Have you been part of any Leadership and Management support (When, where, duration of the ‘support’/course)? Which one did you find most valuable?
- What support do you feel you still need as managers/leaders?
  - - including in communication skills, team building, handling conflicts, supportive feedback and building and maintaining staff motivation
