# Supplementary material for: Prolonged health worker strikes in Kenya- perspectives and experiences of frontline health managers and local communities in Kilifi County
Source: Int J Equity Health. 2020 Feb 10;19:23. doi: 10.1186/s12939-020-1131-y (PMC7011250; doi:10.1186/s12939-020-1131-y)
Supplement: Supplementary file 2 — Additional file 2. Topic guide for focus group discussion-community representatives. [file 12939_2020_1131_MOESM2_ESM.docx]

**Additional file 2: TOPIC GUIDE FOR FOCUS GROUP DISCUSSION-COMMUNITY REPRESENTATIVES**

**Quick reminder about the consent form if that was shared in advance:**

- We are interested in the range of challenges faced by those running the health system, including what factors contribute to making organisations ‘resilient’, or able to withstand and even develop positively in a context of constant stresses (like understaffing) and sudden shocks (like disease outbreaks or big policy changes).
- We are currently trying to learn more about the community’s experience of the health system, especially since devolution
- We’d really value an opportunity to learn from you given your role and experience as a community representative member;
- If you don’t mind, we’d like to record – only use information in general ways (show eg of policy brief)

**A bit about the interviewees: Which locations are they from, how long have they been community representatives?**

Overall before we go on to talk about some of the challenges in relation to the **health system since devolution, what have been some of the strengths/plusses to highlight from the perspective of local communities?** Anything you’d like to share with us about some of the post devolution benefits/achievements in relation to health care?

- *eg who is in the health system, apparent availability of resources or quality of services, ability to question/influence members of the health systems*

Please tell us about some of what you think have been **the most significant post devolution challenges** for local communities in relation to the public health system (eg government hospitals, health centres, dispensaries)? ….

- *eg who is in the health system, apparent availability of resources or quality of services, ability to question/influence members of the health systems*
- For any stand out challenges (that are not about the strike)… how did local communities:
  - Hear about/experience this challenge
  - Cope with it? What did they do/change in response, and why
  - For any changes, was this just temporary while the problem was going on or will it go on for the longer term – positive/negative?

**Re the strikes.**

- Can you remember the different strikes last year and this one? Who it involved and for how long? What do you think led to the strikes?
- Why do you think this nurses’ strike went on so long? 5 months?
- In local communities, how was the nurses strike felt/experienced? Eg what facilities/services were able to keep going (and how?), which ones closed?
- Did community members do anything to try to keep services up and running? What and how?
  - Eg community leaders
  - Members of County Assembly
  - Community Health Workers
  - General public
- What has been the impact of the strikes – and especially the most recent nurses one on local communities?
  - Eg Patient/public access to prevention services? To health care? Ease of access? Quality? The costs they incur? Does this ‘hit’ some households more than others? Who and in what way? (depends on wealth, type of illness/service needed? Other? Gender? A combination?) e.g affordability and use
  - **Any particular impact on children with malnutrition as far as you know? What and how?**
  - Amount of use of healers? Private facilities? Simply staying at home? Why/how and with what impact?
  - Eg health workers and managers’ – how they feel, their motivation? How much support and trust the community have of them? Does this support/trust differ for different cadres/levels
- What will be the immediate changes, and longer term ones, now that the nurses have gone back to work?
  - Do you think the negative impacts will now be over now? Why and how?
  - Any positive imapcts of the strikes – will they continue?
- What has given community members strength/encouragement/support in dealing with challenges associated with the strikes?

**Anything else you’d like to share with us? Ask us?**
